# The selection of matching donors for patients in fecal microbiota transplantation

**DOI:** 10.3389/fmicb.2026.1859411

**Published:** 2026-06-09

**Authors:** Xueyi Qian, Yao Wu, Wei Wang, Huimin Shao, Zhenyu Xu

**Affiliations:** 1 Precision Medicine Center, The First Affiliated Hospital of Wannan Medical University (Yijishan Hospital of Wannan Medical University), Wuhu, China; 2 School of Pharmacy, Wannan Medical University, Wuhu, China; 3 Department of Gastroenterology, The First Affiliated Hospital of Wannan Medical University (Yijishan Hospital of Wannan Medical University), Wuhu, China

**Keywords:** donor screening, donor-recipient matching, fecal microbiota transplantation, gut microbiota, precision medicine

## Abstract

Fecal microbiota transplantation (FMT) is an emerging therapeutic strategy with potential applications in the treatment of various diseases, particularly those associated with gut microbiome dysbiosis. However, clinical trials have demonstrated considerable variability in FMT efficacy—even among patients with the same disease. The heterogeneity of gut microbiota from donors is considered a key factor influencing patient outcomes. Consequently, the development of donor-recipient matching models has emerged as an advanced approach to enhance the effectiveness of FMT. As a practical clinical intervention, the therapeutic impact of FMT on specific diseases requires further investigation. This article reviews the development of donors and the matching patterns between donors and recipients, and summarizes the key factors influencing the transfer of the microbiota. It provides new insights for exploring novel and effective donor-recipient matching patterns.

## 1 Introduction

The human digestive system harbors approximately one trillion microorganisms, including bacteria, viruses, archaea, fungi, protozoa, as well as their bioactive metabolites. They play diverse and essential roles in regulating human physiological and pathological processes, such as facilitating nutrient digestion, reinforcing host defense against pathogens, and maintaining intestinal homeostasis (Bull and Plummer, 2014). The establishment of a normal gut microbiota community has been influenced by multiple factors, including age, gender, specific microorganisms, dietary habits, lifestyle, and other unknown factors (Yatsunenko et al., 2012; Rinninella et al., 2019; Strasser et al., 2021). And this process usually begins in early childhood. An increasing number of clinical studies have shown that the disrupted gut microbiota microenvironment was manifested in patients with inflammatory bowel disease (IBD), *Clostridium difficile* infection (CDI), obesity, Alzheimer’s disease, and other diseases (Zhang et al., 2024; Garza-González et al., 2019; Han et al., 2024; Zhuang et al., 2020). This common phenomenon suggests that the gut microbiota may be related to the pathogenesis of intestinal and extraintestinal diseases. Fecal microbiota transplantation (FMT), referred to as intestinal microbiota transplantation, is an emerging therapeutic strategy that involves transferring gut microbiota isolated from the feces of a healthy donor into the gastrointestinal tract of a recipient with disease (Vindigni and Surawicz, 2017). The primary objective of FMT is to correct intestinal dysbiosis and restore ecological homeostasis within the gut microbiota. In recent years, due to the significant efficacy of FMT in treating recurrent *Clostridium difficile* infections (rCDI), FMT as a novel intervention has gained increasing attention among clinical doctors worldwide. Guidelines suggest that FMT treatment can prevent multiple relapses of *Clostridium difficile* infection (CDI), with a cure rate close to 90% (Cheng and Fischer, 2023). In addition to CDI, IBD has recently been identified as a potential therapeutic target for FMT. However, the efficacy of FMT in IBD varies across studies, and donor specificity is considered by researchers to explain this particular phenomenon (Paramsothy et al., 2017; Caldeira et al., 2020; Zhang et al., 2023). This perspective has been substantiated by a clinical study demonstrating that patients who received FMT batches derived from the same donor exhibited a higher remission rate (37%) compared with those who received material from different donors (18%) (Paramsothy et al., 2017). Furthermore, the existence of “super donors” represented a noteworthy phenomenon discovered in clinical studies of FMT, which was the potential correlation between the success of FMT in treating patients’ diseases and donor selection (Wilson et al., 2019).

Due to the ability of gut microbiota to influence distant organs and related biological pathways through microorganisms or metabolites, it is considered an important endocrine organ by some scholars (Wang et al., 2024). Given the variability in donor microbiota composition and its potential impact on clinical outcomes, optimizing the matching strategy between donors and recipients may represent an advanced approach to address the heterogeneity in the clinical efficacy of FMT. Although numerous clinical trials have demonstrated the therapeutic potential of gut microbiota transplantation in treating various diseases, the diversity among donors introduces significant variability in treatment outcomes, which often falls short of the expectations of both clinicians and patients.

This review synthesizes recent advances in donor selection strategies for FMT in clinical practice and discusses the selection of key characteristics of the optimal donor. Furthermore, we systematically evaluate established and emerging donor–recipient matching approaches and critically analyze the biological, clinical, and logistical factors that inform effective matching, guidance to support rational donor selection in clinical settings.

## 2 Donor selection in FMT development

The use of human feces, known as “yellow soup,” to treat patients with severe diarrhea dates back 1700 years to ancient Chinese medical texts, where it was regarded as the earliest documented precursor of FMT (Zhang et al., 2012). Due to the absence of more reliable historical records, the donor–recipient relationship in these early applications of “yellow soup” remains undetermined. In modern medicine, American surgeon Ben Eiseman and his colleagues reported stool transplantation in four patients with severe pseudomembranous colitis in 1958 (Eiseman et al., 1958). Healthy family members of the patients were selected as donors following informed consent from physicians, patients, and their families. And donor feces were processed into fecal suspension for enema administration, successfully treating three patients with severe pseudomembranous colitis. In the process of implementing FMT, considering safety issues and practical barriers related to allogeneic FMT, some studies have explored autologous fecal microbiota transplantation (aFMT). Notably, a study investigating gut microbiota recovery after antibiotic exposure reported that aFMT significantly accelerated post-antibiotic microbiota reconstitution in six participants (Suez et al., 2018). In addition, a study suggests that autologous FMT collected during the weight loss phase and administered during the recovery phase may help maintain weight loss and improve blood glucose control, and is associated with different microbiome characteristics (Rinott et al., 2021). In a preliminary trial of adults with mild-to-moderate ulcerative colitis (UC), pooled-donor FMT achieved the primary outcome in 12 of 38 participants (32%), versus 3 of 35 (9%) with autologous FMT (Costello et al., 2019). These studies indicate that patients themselves can also serve as donors of fecal microbiota, with a slight improvement effect on their intestinal environment.

Selecting family members, friends, or the patient themselves as fecal donors imposes a significant logistical burden on the treatment team and could delay FMT delivery from prescription to administration. The fecal bank is a centralized facility used for screening donors, processing, and storing FMT preparations. Supplying these preparations to clinical doctors and researchers can improve clinical treatment efficiency and make it easier to monitor their safety and efficacy (Panchal et al., 2018; Kim and Gluck, 2019). In 2015, the Chinese Fecal Microbiota Bank was established and used microbiota preparations from qualified healthy donors for remote FMT treatment (Dai et al., 2019). Eighteen critically ill patients with antibiotic-associated diarrhea (AAD) received salvage FMT from China’s FMT Bank, and favorable clinical outcomes were observed. In 2019, FMT experts from Europe, North America, and Australia reached consensus on donor screening criteria at the International Consensus Conference on Stool Banking for FMT (Cammarota et al., 2019). Young adults (aged <50 or <60 years following appropriate colorectal cancer screening) were prioritized as potential donors, with no gender preference.

The recruitment of fecal microbiota transplant donors is a challenging process, as only a few candidates meet the eligibility criteria after rigorous screening. Data from a large fecal bank in the United States showed that nearly 90% of donor candidates were excluded after medical evaluation (Zhang et al., 2022). A fecal bank in Australia also provided similar data, with only 12 out of 116 potential donors (10%) selected as study donors (Paramsothy et al., 2015). The qualification rate of donor screening in the Chinese microbiota database was even lower (Dai et al., 2019). Although building a donor library can provide more convenient gut microbiota for the clinical treatment of FMT, the low efficiency of donor screening has increased the difficulty of clinical application of FMT.

**Figure 1:**
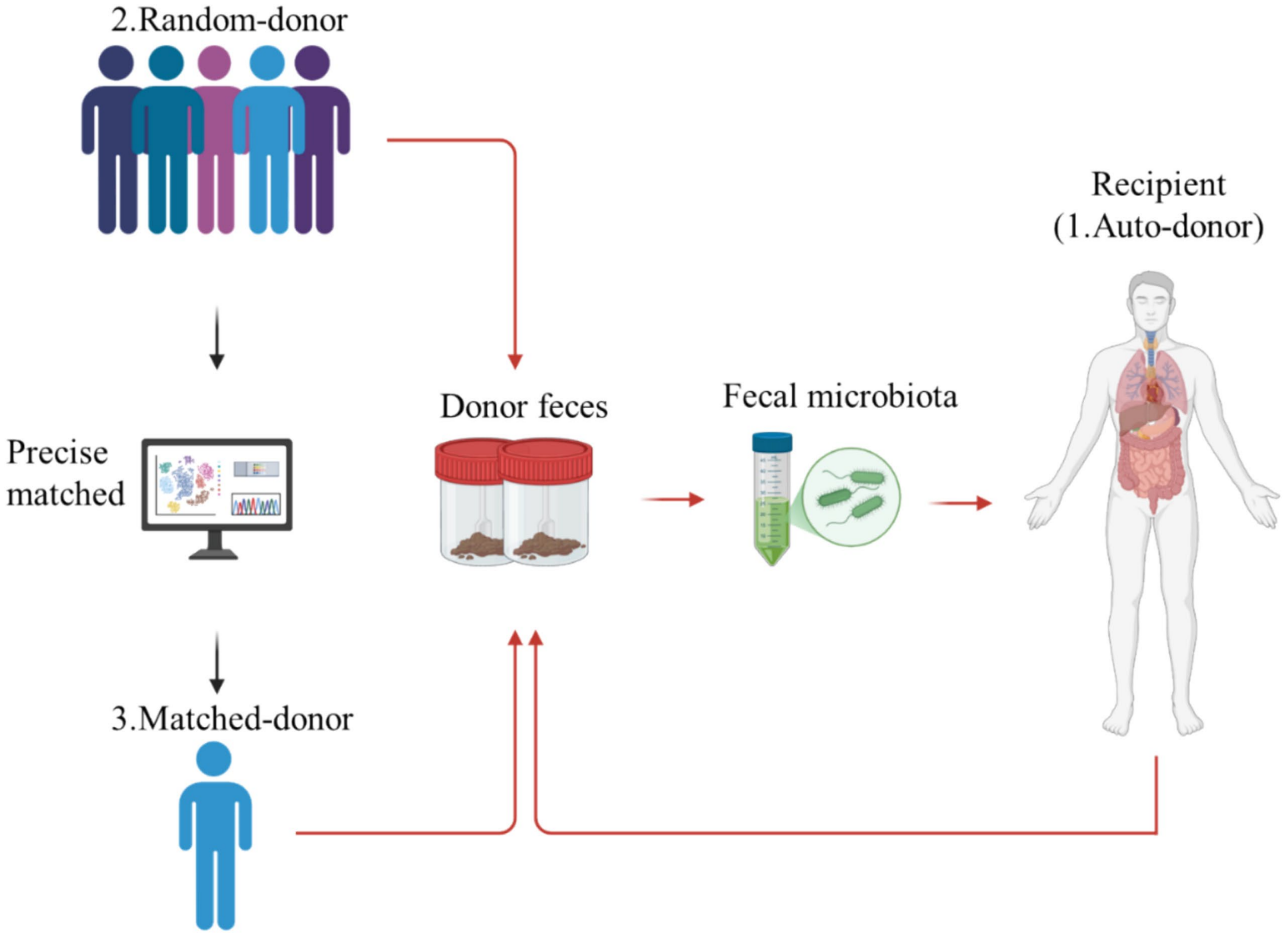
Donor selection of patients. (1) Auto-donor for patient; (2) Random-donor for patient; (3) Matched-donor for patient. The black arrow represents the screening process for a matched donor. The red arrow indicates a brief process of fecal donation from the donor and subsequent microbiota transplantation. Created in BioRender. Qian, X. (2026) https://BioRender.com/2pu5215.

These studies indicated that donor selection was a pivotal factor governing both the efficacy and safety of FMT. Beyond auto-donor and random-donor sourced from public FMT banks selected via standardized health screening, researchers are advancing evidence-informed donor–recipient matching strategies, integrating microbial, immunological, and clinical profiling, to improve therapeutic response rates and broaden the scope of FMT applications (Figure 1).

## 3 Donor–recipient matching

Gut microbiota modulation via FMT has demonstrated promising therapeutic efficacy in clinical trials for multiple conditions, including ulcerative colitis, recurrent *Clostridioides difficile* infection, and autism spectrum disorders (Costello et al., 2019; Juul et al., 2025; Chen et al., 2024). However, the effectiveness of FMT treatment varies greatly in different studies (Paramsothy et al., 2017; Caldeira et al., 2020). There are many reasons for this phenomenon, among which donor recipient matching is widely recognized as a key determinant of treatment success. Consequently, the development of evidence-based, individualized donor matching strategies represents a key frontier in FMT research.

A donor–recipient matching model was established through meta-analytic integration and applied to inform evidence-based donor selection for ulcerative colitis (UC) in FMT (Zhang et al., 2023). In this model, six key microbiome indicators—α-diversity, Bray-Curti’s distance, beneficial and pathogenic bacterial taxa, and beneficial versus pathogenic metabolic pathways—form the foundation of an Analytic Hierarchy Process (AHP) framework for donor–recipient evaluation and treatment response prediction. Moreover, donor–recipient matching could be stratified according to enterotype concordance. CDI and IBD patients consistently exhibit two dominant enterotypes: RCPT/E, enriched in Enterobacteriaceae, and RCPT/B, enriched in Bacteroidetes (He et al., 2022). Donors were similarly stratified into two enterotypes—DONOR/P (Prevotella-dominant) and DONOR/B (Bacteroides-dominant). Notably, RCPT/E recipients achieved the highest clinical response rate (70.88%) in this model. In another study on the treatment of symmetric polyneuropathy in patients with diabetes by FMT, better treatment outcomes were often demonstrated in patients who had a match between the transplant and the recipient (Yang et al., 2023). These studies suggest that determining the enterotypes of donor and recipient and matching the donor and recipient before FMT treatment is an effective method to improve the curative effect method for patients.

An interesting phenomenon had emerged in clinical studies of gut microbiota transplantation: the super-donor phenomenon (Wilson et al., 2019). This phenomenon was first reported in a clinical randomized controlled trial, in which 7 out of 9 IBD patients in the FMT treatment group who achieved remission received stool from the same donor (Moayyedi et al., 2015). This inevitably evoked the notion of a “super donor” effect. Unfortunately, the research team did not provide a deeper analysis. A natural “super-donor” consortium, a microbial community, was discovered in a mouse study, which consortium robustly engrafted into diverse recipients and resisted reciprocal colonization (Urtecho et al., 2024).

FMT donor selection emphasized donor phenotypes while overlooking recipient-specific determinants. To bridge this gap, some researchers reintegrated predictive microbial biomarkers with a genetic algorithm (GA), leveraging recipient gut microbiota dynamics. Specifically, these dynamics include compositional shifts before and after transplantation, which helps identify the optimal donor for each patient (Shtossel et al., 2023). On the other hand, the mismatch in the microbial community caused by microbial transplantation could lead to persistent non-targeted metabolic and immune regulatory effects (DeLeon et al., 2026). Collectively, these findings indicated that the selection of donors whose microbiota were compatible with the recipient’s pre-existing genetic profile—may be a critical determinant of therapeutic success in microbiota-based interventions.

## 4 Factors of donor–recipient matching

As clinical evidence for FMT continues to accumulate, it has become increasingly clear that FMT entails the transfer of a donor’s entire intestinal microbial ecosystem to the recipient. Consequently, multidimensional donor–recipient matching, which is grounded in the complexity of the gut microbiota, interindividual host variation, and disease-specific pathobiology, represents a pivotal advancement in establishing FMT as a safe, effective, and reproducible therapeutic modality.

### 4.1 Microbial species diversity

The human gastrointestinal tract hosts a highly complex and dynamic microbial ecosystem comprising bacteria, archaea, fungi, viruses, and their associated metabolites (Qin et al., 2010). Quantifying alpha diversity, the within-sample measure of microbial community richness and evenness, is essential for characterizing community structure and assessing ecological stability. In contrast, beta diversity defined as the compositional dissimilarity between distinct microbial communities is commonly estimated using metrics that account for both shared and unique taxa across samples (Lozupone and Knight, 2008). Gut microbiota dysbiosis and reduced microbial alpha diversity are well-established contributors to a range of pathological conditions, including type 2 diabetes, obesity, inflammatory bowel disease, autoimmune disorders, as well as other systemic diseases (Karimi et al., 2024). In patients with inflammatory bowel disease, both the taxonomic richness and phylogenetic diversity of the gut microbiota were significantly diminished (Hu et al., 2022). Following FMT in IBD patients, longitudinal improvements in clinical outcomes correlated strongly with increases in alpha diversity and robust engraftment of donor-derived microbial taxa (Zhang et al., 2024). Notably, certain disease contexts exhibited paradoxical elevations in microbial diversity relative to healthy controls. For instance, a study in multiple myeloma reported significantly higher bacterial species-level alpha diversity in newly diagnosed and relapsed patients compared with age- and sex-matched healthy individuals (Zhu et al., 2024). These findings indicated that microbial species diversity served not only as a structural indicator of community composition but also as a functional biomarker correlated with host health status and physiological homeostasis.

### 4.2 Special species

Probiotics confer beneficial effects through two primary mechanisms: firstly, by augmenting the abundance of commensal bacteria in the intestinal tract via their own proliferation (Fassarella et al., 2021); secondly, by inhibiting the colonization and expansion of pathogenic bacteria through competitive exclusion (Monteagudo-Mera et al., 2019). For instance, in a defined probiotic consortium containing *Akkermansia muciniphila* and *Clostridioides difficile*, *Akkermansia muciniphila* occupies a critical ecological niche and functions as a competitive inhibitor—displacing pathogens via the secretion of mucin-derived polysaccharides (Pereira et al., 2020). *Clostridium difficile*, with particular attention to the expression of RT027 gene associated with toxins, should be screened as a priority in donor selection (Wang et al., 2026). Furthermore, bacterial biopolymers synthesized by *Bifidobacterium* and *Lactobacillus* species act as fermentable carbon substrates for the broader gut microbiota, thereby supporting microbial antagonism against pathogens and promoting a resilient, structurally stable intestinal ecosystem (Castro-Bravo et al., 2018). *Prevotella,* a genus comprising over 50 validated species, is a ubiquitous and functionally versatile component of the human gut microbiome; its interspecies ecological interactions are increasingly recognized as key determinants of its metabolic influence on host physiology and health outcomes (Tett et al., 2021). Critically, drug pharmacokinetics and pharmacodynamics are modulated by specific gut microbial taxa through the alteration of drug metabolism pathways. Integrated multi-omics analyses revealed that nucleotide biosynthesis pathways mediated by prevalent *Bacteroidetes* species were correlated with resistance to neoadjuvant chemoradiotherapy in patients with locally advanced cancers (Teng et al., 2023). Likewise, members of the phyla *Proteobacteria* and *Firmicutes* enzymatically convert 5-fluorouracil into its inactive metabolite, dihydrofluorouracil, which effectively recapitulates a major host-mediated detoxification route. In contrast, *E. coli* perturbs endogenous pyrimidine metabolism, potentially compromising nucleotide homeostasis (Spanogiannopoulos et al., 2022). Notably, murine models colonized with either *Methanobrevibacter rupellensis* or *E. coli* engineered to heterologously express the myo-inositol–catabolizing iolG gene demonstrate significantly enhanced intestinal lipid absorption, culminating in diet-independent obesity phenotypes (Wu et al., 2024). Although the relative abundance of these functionally influential microbes may be low, and while microbiota-targeted interventions may induce only subtle compositional shifts, their biochemical activities and keystone ecological roles exert disproportionate influence on therapeutic outcomes. However, gut microbiota transplantation research has largely overlooked the identification and functional validation of microbial taxa whose presence, activity, or metabolic signatures are causally linked to clinical efficacy.

### 4.3 Age

The composition and functional profile of the gut microbiota undergo dynamic, age-associated changes (López-Otín et al., 2023). During early life, the gut microbiota plays a pivotal role in host immune maturation, metabolic programming, and neurodevelopment. Breastfeeding critically supports neonatal and infant health, which not only by delivering essential nutrients and immunomodulatory factors but also by actively shaping the establishment and succession of a resilient microbial community (Ahrens et al., 2024). Microbial diversity increases progressively during childhood and typically reaches a relatively stable, individualized configuration in healthy adulthood. In contrast, aging is associated with progressive dysbiosis, characterized by reduced taxonomic richness, decreased functional redundancy, and diminished resilience, which collectively contribute to a decline in overall ecological stability and diversity (Ghosh et al., 2022). Consequently, donor–recipient age disparity may constitute a biologically relevant variable influencing the engraftment efficiency, functional restoration capacity, and clinical outcomes of FMT. Preclinical studies demonstrate that heterochronic FMT, which involves the transfer of microbial communities from young donors to aged recipients, can attenuate age-associated vascular dysfunction and partially restore metabolic homeostasis (Cheng et al., 2024). Conversely, transplantation of fecal microbiota from aged donor mice into young recipients induces hippocampal synaptic deficits, impairing spatial learning and memory performance by dysregulating proteins related to both synaptic plasticity and neurotransmission (D'Amato et al., 2020).

Multiple studies in centenarians have consistently reported a decline in core, highly abundant bacterial taxa, such as *Bacteroide*s and *Roseburia*, concomitant with an enrichment of specific genera, including *Bifidobacterium* and *Akkermansia*, suggesting a potential association between these microbes and extended health-span or lifespan (Biagi et al., 2016). Preclinical evidence further supports this notion: FMT from wild-type young-adult mice significantly improved both health-span and longevity in mouse models of premature aging; notably, supplementation with *Verrucomicrobia* or *Akkermansia* alone recapitulated key beneficial effects. Contrastly, FMT from elderly donors into healthy wild-type recipients has been shown to induce adverse metabolic alterations (Bárcena et al., 2019). Clinical trials of FMT in pediatric populations, including those targeting inflammatory bowel disease (IBD; NCT03399188), autism spectrum disorder (NCT03426826), ulcerative colitis (NCT03582969), Crohn’s disease (NCT03378167), and refractory Clostridioides difficile infection (NCT02134392), have largely employed adult donors, yet donor age is frequently reported only as “adult” or remains unreported in trial registries. Both the North American Society for Pediatric Gastroenterology, Hepatology, and Nutrition (NASPGHAN) and the European Society for Pediatric Gastroenterology, Hepatology, and Nutrition (ESPGHAN) emphasize that rigorous investigation into age-matched donor–recipient pairing is warranted to establish the long-term safety and therapeutic efficacy of pediatric FMT (Davidovics et al., 2019). Accordingly, age-matched donor selection may mitigate potential risks, including aberrant neurodevelopment, immune dysregulation, or early-onset chronic disease, associated with the transfer of age-incongruent microbial communities (MacLellan et al., 2021).

### 4.4 Gender

Gender is a biologically and clinically significant factor in the pathogenesis of several diseases, including IBD (Andersen et al., 2024). Consequently, emerging evidence underscores the importance of incorporating sex as a key variable in donor selection criteria for FMT, with the aim of optimizing therapeutic efficacy and safety (Benítez-Páez et al., 2022). Post-pubertal sex differences, which are mediated in part by sex hormones, exert robust and persistent influences on gut microbial community structure and function (Kim et al., 2020). Notably, the bacterial-to-human cell ratio exhibits sexual dimorphism: it averages 2.2 in adult females versus 1.3 in adult males (Sender et al., 2016). A large-scale cohort study involving 2,338 Han Chinese adults (aged 26–76 years) further confirmed sex-specific patterns in gut microbiota composition; specifically, premenopausal women demonstrated significantly higher alpha diversity compared with age-matched men (Zhang et al., 2021). Mechanistically, Di Li et al. isolated *Mycobacterium neoaurum* from male patients with major depressive disorder; this strain encodes 3β-hydroxysteroid dehydrogenase (3β-HSD), an enzyme implicated in testosterone catabolism. In murine models, colonization with *M. neoaurum* reduced both systemic and central nervous system testosterone levels and induced depression-like behavioral phenotypes (Li et al., 2022). Complementarily, the same group identified *Klebsiella pneumoniae*, a strain capable of estradiol degradation, in fecal samples from premenopausal women with depression. Furthermore, gavage of mice with *E. coli* engineered to express 3β-HSD resulted in decreased serum estradiol concentrations and concomitant depression-like behaviors (Sender et al., 2016). Operational data from BiomeBank (South Australia) indicate that, on average, male donors contributed significantly more FMT doses per collection session than female donors (Tucker et al., 2023). Additional experimental evidence reveals that ischemic stroke induces a masculinization of the gut microbiota in female mice, whereas reciprocal FMT via oral gavage attenuates sex-specific microbial divergence, rendering male and female recipients’ microbiomes more phenotypically convergent (Wang et al., 2022). Similarly, sex-dependent transfer of radiation sensitivity has been demonstrated following FMT (Cui et al., 2017). Collectively, these findings highlight the potential physiological consequences of donor–recipient sex mismatch in FMT, particularly with respect to endocrine homeostasis and sex hormone–regulated pathways.

### 4.5 Body mass

A recent study integrating DNA methylation profiling and 16S rRNA gene sequencing of gut microbiota from 342 individuals with body mass index (BMI) ranging from 18 to 40 kg/m^2^ identified a statistically significant negative association between relative abundance of *Ruminococcus* and BMI. Notably, mediation analysis revealed that methylation levels at the MACROD2/SEL1L2 differentially methylated region (DMR) accounted for approximately 19% of the total effect of *Ruminococcus* abundance on BMI (Salas-Perez et al., 2023). In contrast, no significant difference was observed in the *Firmicutes*-to-*Bacteroidetes* (F/B) ratio between lean and overweight/obese male college students in China compared with their normal-weight peers. However, linear discriminant analysis coupled with effect size measurements (LEfSe) indicated that *Blautia*, *Anaerotruncus*, and their uncultured relatives were significantly enriched in the lean group (LDA score ≥ 3), whereas *Parabacteroides* and its uncultured relatives were selectively enriched in the overweight/obese group (Lv et al., 2019). Clinical evidence further suggests that donor anthropometric characteristics, including body weight and metabolic status, may be partially transferred to recipients via FMT. For instance, a female patient with recurrent *Clostridioides difficile* infection developed new-onset obesity following FMT from a healthy but overweight donor (Alang and Kelly, 2015). Similarly, in a clinical trial of FMT for CDI, recipients who received microbiota from donors with obesity and metabolic syndrome exhibited a significant increase in insulin resistance (Singh et al., 2014). Conversely, patients with metabolic syndrome demonstrated improved insulin sensitivity after receiving FMT from lean donors (Kootte et al., 2017). In a separate interventional study involving obese, non-diabetic adults (BMI ≥ 24 kg/m^2^), three biweekly FMT administrations over 12 weeks resulted in ≥5% weight loss from baseline in 52% of participants classified as “responders” (Ruan et al., 2025). Nevertheless, findings across studies remain inconsistent: interventions using microbiota from donors who had previously undergone substantial weight loss induced measurable shifts in recipient gut microbial composition but failed to produce clinically meaningful improvements in body weight or insulin sensitivity. Despite this heterogeneity, donor BMI is currently retained as an initial screening criterion in most FMT donor selection protocols, particularly extremes of underweight and overweight.

### 4.6 Enterotypes

Enterotypes were first proposed by the Peer Bork group in 2011, based on cross-cohort analyses of human gut microbiota composition. Three robust, geographically invariant clusters—predominantly distinguished by the relative abundance of *Bacteroides, Prevotella,* and *Ruminococcus*—were consistently identified across multiple independent datasets (Arumugam et al., 2011). However, only the *Bacteroides*- and *Prevotella*-enriched enterotypes exhibited reproducible taxonomic and functional signatures; the *Ruminococcus*-associated cluster lacked consistent defining features and remains incompletely characterized. Subsequent work by Liang et al. reported that fecal-derived enterotypes were primarily driven by *Bacteroidetes*, *Prevotella,* and *Enterobacteriaceae*, with significant correlations observed between these profiles and long-term dietary patterns (Liang et al., 2017). Functionally, enterotypes are associated with divergent metabolic capacities—particularly in the degradation of dietary substrates—and consequently differ in their production profiles of short-chain fatty acids (SCFAs), which may modulate host energy homeostasis and immune regulation (Christensen et al., 2018). Notably, *Enterobacteriaceae*- and *Bacteroides*-enriched enterotypes are overrepresented in patients with CDI and IBD. Moreover, clinical outcomes following FMT in these patient populations correlate significantly with both donor–recipient enterotype congruence and overall microbial community similarity (He et al., 2022). To date, no universally accepted framework for enterotype classification exists. In UC, an integrative analysis of 11 independent 16S rRNA gene sequencing datasets enabled the identification of three distinct enterotypes—*Bacteroidetes*-dominant (ET-B), *Lachnospiraceae*-dominant (ET-L), and *Clostridiaceae*-dominant (ET-C)—using hierarchical clustering, supervised machine learning, and species co-occurrence network (SCN) modeling (Wu et al., 2024). In oncology, two functionally distinct gut microbiota configurations—termed “favorable” and “unfavorable”—have been linked to differential responses to immune checkpoint blockade (e.g., anti-PD-1/PD-L1 therapy); notably, FMT from donors with a favorable configuration significantly improved therapeutic response rates in refractory melanoma patients (Hu et al., 2025). Although enterotype-based stratification has not yet been prospectively validated in large-scale randomized clinical trials, accumulating evidence underscores substantial inter-individual variation in gut microbial ecology. This heterogeneity supports the rationale for incorporating enterotype profiling into donor–recipient matching algorithms to refine FMT personalization and improve predictive accuracy of treatment efficacy.

### 4.7 IgA-coated bacteria

IgA-coated bacteria in the intestinal microbiota contribute to colonization resistance and immune homeostasis in healthy individuals by impeding microbial translocation and preserving the integrity of the intestinal epithelial barrier (DuPont et al., 2022). During FMT, IgA-coated bacteria are effectively transferred from donors to recipients while retaining their immunoglobulin A coating (Lima et al., 2022). In a clinical study involving 20 patients with ulcerative colitis who received FMT from two healthy donors (Chu et al., 2021), no significant difference was observed in the relative abundance of IgA-coated fecal bacteria between clinical responders and non-responders—either pre-FMT or at 4 weeks post-FMT. However, the IgA-coated bacterial community in the four-week post-FMT cohort exhibited significantly higher alpha diversity compared with baseline. Notably, donors and their respective recipients shared 29 IgA-coated bacterial operational taxonomic units (OTUs) or amplicon sequence variants (ASVs). Furthermore, in a murine model of dextran sulfate sodium (DSS)-induced colitis characterized by heightened susceptibility to intestinal inflammation, FMT enriched with IgA-coated bacteria conferred robust protection against disease development (Gupta et al., 2019). Collectively, these findings indicate that both the compositional diversity and donor–recipient congruence of IgA-coated bacterial populations may be critical determinants of FMT therapeutic efficacy.

### 4.8 Bacteriophage

The viral component of the human gut microbiota is predominantly composed of bacteriophages, which play crucial roles in shaping microbial composition, promoting bacterial diversity, and facilitating horizontal gene transfer (Sutton and Hill, 2019). In most cases, bacteriophages can be classified into families based on tail morphology: *Siphoviridae*, *Myoviridae*, and *Podoviridae* (Shkoporov and Hill, 2019). Fecal samples from different human donors harbor unique combinations of dozens of phage variants belonging to these three families (Castro-Mejía et al., 2015; Hoyles et al., 2014). In clinical FMT studies, donor-derived viruses are readily detectable in recipients, with up to 32 distinct donor-associated viral taxa identified in recipient samples, indicating efficient viral transfer via FMT (Chehoud et al., 2016). Following FMT, individuals with recurrent *Clostridioides difficile* infection (rCDI) exhibit a shift in gut phage profiles toward donor-like phage communities, accompanied by increased phage alpha diversity; however, the extent of donor phage engraftment varies depending on recipient-specific factors (Ji et al., 2025). Moreover, phage-mediated therapies hold promise for overcoming donor variability and safety concerns associated with conventional FMT (Rasmussen et al., 2024). In preclinical colorectal cancer (CRC) mouse models, the *Bacteroides fragilis*–targeting phage VA7 selectively inhibits *B. fragilis* growth and restores chemosensitivity (Ding et al., 2025). These findings indicate that bacteriophages contribute to determining FMT efficacy. The next generation of microbiota-based therapeutics may involve targeted bacterial or viral interventions—not necessarily whole-feces transplantation.

### 4.9 Immunity

The intestinal immune system must respond appropriately to billions of microorganisms in the intestine, while providing active immunity against invading pathogens. This disruption of balance may lead to intestinal diseases, including inflammatory bowel disease, Crohn’s disease, and ulcerative colitis (Mörbe et al., 2021).

A 5-year follow-up data of more than 8,000 FMT patients showed a clear disease-specific pattern in the immune related efficacy of FMT (Wang et al., 2025). The highest efficacy is observed in acute intestinal inflammatory diseases (mainly rCDI), with a success rate of over 90%; The response rate in functional intestinal diseases exceeds 60%; The response rate is less than 60% in organic intestinal diseases or chronic intestinal inflammatory diseases; In extraintestinal diseases (including autoimmune diseases), the response rate is less than 50%. This result suggests that the therapeutic effect of FMT on patients’ diseases is closely related to the immune characteristics of the disease itself and the degree of microbiota immune axis dependence. Although fecal microbiota transplantation cannot directly provide donor antibodies to patients, the new microbiota can undoubtedly alter the patient’s immune characteristics. A study on a mouse model of food allergy induced by ovalbumin showed that FMT treatment can significantly improve gut microbiota structure, reduce levels of Th2 related inflammatory factors, decrease the number of mast cells and eosinophils, and inhibit the production of IgE and OVA specific antibodies (Huang et al., 2024). Furthermore, in patients with recurrent Clostridioides difficile, the expression of IL-33 and type 2 immune EGFR family ligand amphiregulin in colonic epithelium significantly increased after FMT treatment (Moreau et al., 2026). These research results indicate a correlation between donor microbiota and patient immune progression, but there is significant heterogeneity in the results. Currently, there are few studies reporting the correlation and related mechanisms between donor immune status, gut microbiota, and patient immune status during FMT treatment. This is also an important research direction in the future and will play a significant role in donor acceptor matching.

## 5 Potential donor selection strategies

The quality of the donor microbiota not only directly influences clinical efficacy but is also closely associated with the risk of adverse events. Therefore, the selection of potential donors is a central determinant of the success and safety of FMT therapy. Further optimize donor screening strategies beyond conventional safety assessments has become a critical issue that urgently needs to be addressed in current FMT research. Based on the preceding review and evidence from the existing literature, we propose two potential donor selection strategies to provide perspectives and directions for future FMT-related research.

The first is retrospective selection. This strategy utilizes outcomes and data from clinical FMT studies to identify “superior donors” that demonstrate higher therapeutic efficacy for specific diseases or particular patient populations. A typical example of this approach is the discovery and conceptualization of the “super-donor” (Wilson et al., 2019). The advantage of retrospective selection lies in its orientation toward real-world clinical outcomes, which provides a strong practical foundation; however, it relies on the availability of high-quality historical data.

The second is prospective selection. In addition to performing traditional donor safety screening, this approach also requires systematic recording and analysis of multidimensional biological characteristics of donors. These characteristics should include microbial species diversity, specific species, age, gender, body mass, enterotypes, IgA-coated bacteria, and bacteriophages. A rational and efficient multi-factor evaluation system could be established using mathematical models or artificial intelligence for donor-recipient matching. Some matching models have been constructed using published data, but lack clinical validation (Zhang et al., 2023; He et al., 2022), which means that such strategies still require more preclinical or clinical studies to confirm.

## 6 Conclusions and prospects

FMT, as a novel therapeutic approach that reconstructs the gut microbiota, has been applied in the treatment of various diseases. During donor selection, safety must be ensured—and adaptability to the recipient should also be considered. This review summarizes the evolution of donor screening criteria, which have shifted from autologous to allogeneic donors in FMT practice.

**Figure 2:**
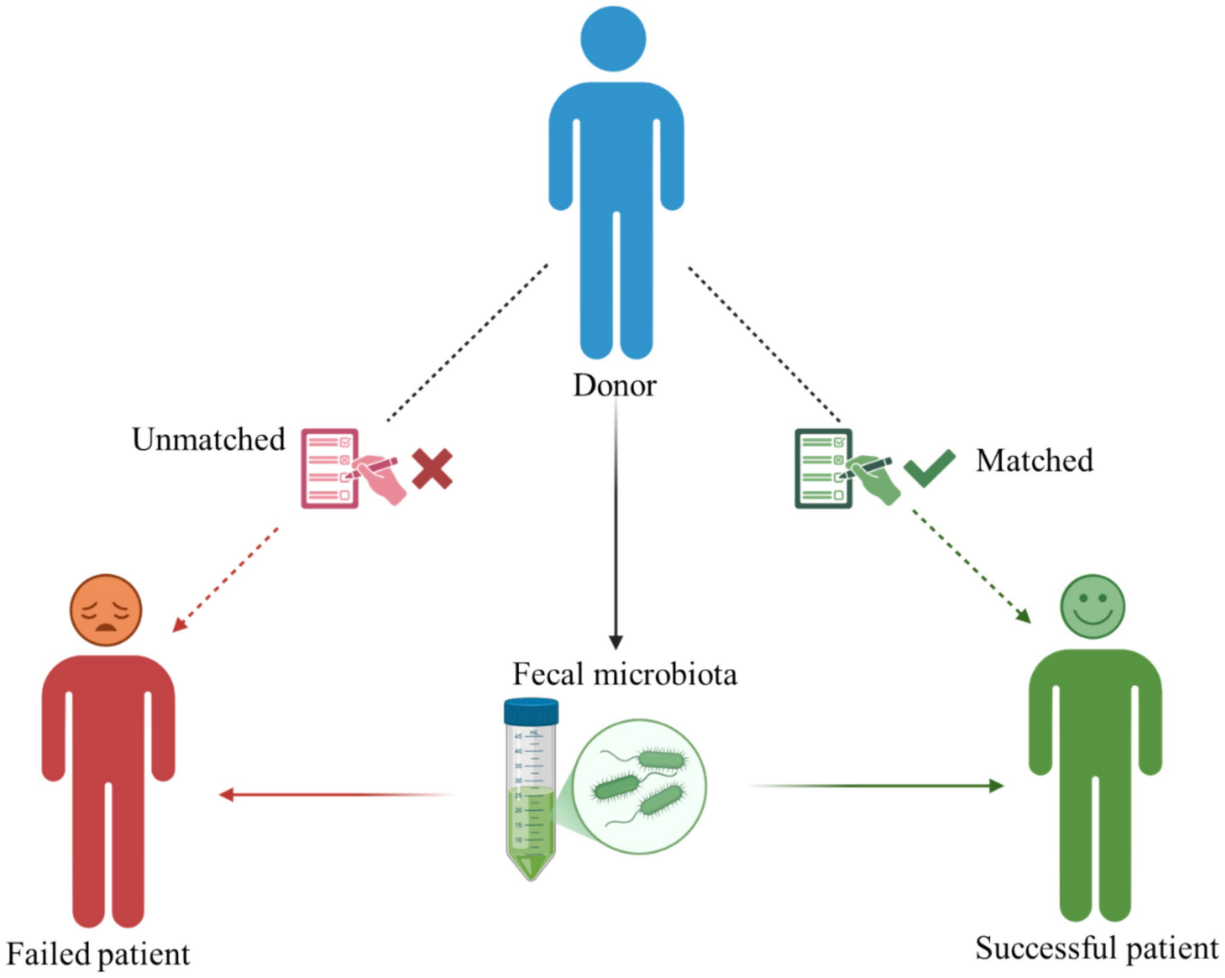
The match or mismatch between donor and patient affects the success or failure of FMT. The dashed line represents the matching process, while the solid line represents the FMT process and predicted treatment outcomes. Created in BioRender. Qian, X. (2026) https://BioRender.com/2pu5215.

In addition, this article systematically reviews donor–recipient matching strategies in FMT and the factors influencing treatment efficacy. Matching strategies are primarily based on structural features of the gut microbiota or enterotype classification. Although these approaches have been validated in retrospective analyses, prospective clinical trials confirming their utility remain lacking. Prior to fully elucidating the complex interrelationships and uncharted domains of the gut microbiota, donor characteristics may serve as key criteria for matched donor selection, including age, gender, Immunity, BMI, enterotype, IgA-coated bacterial profiles, and bacteriophage composition (Figure 2).

In summary, this review comprehensively examines donor–recipient matching approaches and associated characteristics in FMT, and analyzes how multiple donor-derived factors influence treatment efficacy. Further theoretical and clinical research is needed to clarify the pivotal role of donors in FMT and to advance our understanding of the human gut microbiota.
